# A high‐resolution approach for the spatiotemporal analysis of forest canopy space using terrestrial laser scanning data

**DOI:** 10.1002/ece3.4193

**Published:** 2018-06-11

**Authors:** Carsten Hess, Werner Härdtle, Matthias Kunz, Andreas Fichtner, Goddert von Oheimb

**Affiliations:** ^1^ Institute of Ecology Faculty of Sustainability Leuphana University of Lüneburg Lüneburg Germany; ^2^ Institute of General Ecology and Environmental Protection Faculty of Environmental Sciences Technische Universität Dresden Tharandt Germany; ^3^ German Centre of Integrative Biodiversity Research (iDiv) Halle‐Jena‐Leipzig Leipzig Germany

**Keywords:** α‐shape, canopy packing, change detection, crown modeling, multitemporal coregistration, remote sensing, time series, voxel grid

## Abstract

Forest canopies and tree crown structures are of high ecological importance. Measuring canopies and crowns by direct inventory methods is time‐consuming and of limited accuracy. High‐resolution inventory tools, in particular terrestrial laser scanning (TLS), is able to overcome these limitations and obtain three‐dimensional (3D) structural information about the canopy with a very high level of detail. The main objective of this study was to introduce a novel method to analyze spatiotemporal dynamics in canopy occupancy at the individual tree and local neighborhood level using high‐resolution 3D TLS data. For the analyses, a voxel grid approach was applied. The tree crowns were modeled through the combination of two approaches: the encasement of all crown points with a 3D α‐shape, which was then converted into a voxel grid, and the direct voxelization of the crown points. We show that canopy occupancy at individual tree level can be quantified as the crown volume occupied only by the respective tree or shared with neighboring trees. At the local neighborhood level, our method enables the precise determination of the extent of canopy space filling, the identification of tree–tree interactions, and the analysis of complementary space use. Using multitemporal TLS data recordings, this method allows the precise detection and quantification of changes in canopy occupancy through time. The method is applicable to a wide range of investigations in forest ecology research, including the study of tree diversity effects on forest productivity or growing space analyses for optimal tree growth. Due to the high accuracy of this novel method, it facilitates the precise analyses even of highly plastic individual tree crowns and, thus, the realistic representation of forest canopies. Moreover, our voxel grid framework is flexible enough to allow for the inclusion of further biotic and abiotic variables relevant to complex analyses of forest canopy dynamics.

## 1 INTRODUCTION

Many key ecosystem functions and processes in forests are related to the structure and spatiotemporal dynamics of the canopy. Tree canopies exchange carbon, water, and energy with the atmosphere, and they are important for the resistance and resilience of forests to disturbances, govern successional processes, serve as wildlife habitat, and are of great value for biodiversity conservation (Nakamura et al., 2017; Weng et al., 2015). Photosynthesis is the primary function of forest canopies, and in closed‐canopy forests, light is often the key resource for primary productivity. Therefore, trees compete for space in the canopy (Kellner & Asner, 2014). Because tree communities are the sum of co‐occurring individuals, they can be considered as networks of locally interacting individuals (Michalet et al., 2015). The outcome of competition for canopy space is, thus, strongly affected by tree interactions at the local neighborhood level (Fichtner et al., 2018). Furthermore, these tree neighborhood interactions constitute a key factor determining a tree's architecture and, ultimately, growth, and productivity patterns (Fichtner et al., 2017; Potvin & Dutilleul, 2009; Pretzsch, 2014; von Oheimb et al., 2011).

The high ecological importance of tree crowns and canopy structure has resulted in a wealth of studies on crown morphology, plasticity and growth, canopy structures, and crown‐related tree interactions (for recent reviews see Ishii & Asano, 2010 and Pretzsch, 2014).

Crowns and canopies are traditionally measured by direct inventory methods, which are time‐consuming and of limited accuracy when assessing more complex crown characteristics, for example, capturing asymmetries in tree growth. In particular, quantifying crown characteristics of individual trees and their local neighbors with high resolution and spatially explicit with temporal replications have been logistically unfeasible. Thanks to recent technological advances, high‐resolution inventory tools now have the potential to overcome these limitations. Airborne laser scanning (ALS) techniques have been successfully applied to characterize canopy structure (Leiterer, Furrer, Schaepman, & Morsdorf, 2015) and measure essential parameters of individual tree crown characteristics (Jung, Kwak, Park, Lee, & Yoo, 2011). However, ALS is largely restricted to the top canopy layer, as signal occlusion by the upper canopy means that the elements in the lower canopy are recorded in less detail (Jung et al., 2011). Terrestrial laser scanning (TLS) has been established as a complementary approach for the time‐efficient and nondestructive measurement of three‐dimensional (3D) structural elements of trees (Liang et al., 2016). TLS has been applied to analyze standard tree and stand dendrometrics, such as stem diameter and tree height (Li, Hess, von Wehrden, Härdtle, & von Oheimb, 2014; Liang et al., 2016), and more recently to analyze 3D tree topology, canopy structure, and wood volume of branches (Bienert, Hess, Maas, & von Oheimb, 2014; Calders et al., 2015; Hackenberg, Morhart, Sheppard, Spiecker, & Disney, 2014; Hess, Bienert, Härdtle, & von Oheimb, 2015; Kunz et al., 2017; Raumonen et al., 2013).

For the analysis of tree crown metrics, several point cloud‐based methods exist. A common approach is to compute a 2D or 3D α‐shape around a set of crown points to measure crown projection area and crown volume (CV) (e.g., Bayer, Seifert, & Pretzsch, 2013; Fernández‐Sarría et al., 2013; Olsoy, Glenn, Clark, & Derryberry, 2014) as well as more complex metrics such as crown sinuosity and roughness (Martin‐Ducup, Schneider, & Fournier, 2016). α‐shapes are a generalization of the convex hull concept (Edelsbrunner, Kirkpatrick, & Seidel, 1983), and by adjusting the value of α, even small details and concave surface structures in crown morphology can be captured. The rationale for using α‐shapes is often to simplify the point cloud and the amount of data without losing information on occupied crown space (Rutzinger, Pratihast, Elberink, Sander, & Vosselman, 2011). However, oversimplification may lead to the loss of details within the crown, that is, branches that lie within the α‐shape hull. Therefore, sensible α‐values need to be selected depending on the application and quality of the point cloud.

Another popular approach is voxel grids. They fragment the original point cloud into 3D cubes (voxels) with a defined edge length. This can significantly reduce the size of the point cloud by keeping most of the structural information of the tree including its branches. However, similar to the α‐value, the voxel size has to be chosen with care as it can have a significant effect on the measured property, in particular when computing volumes. With respect to tree crowns, voxel grids have been used to compute leaf area index and foliage (Béland, Widlowski, Fournier, Côté, & Verstraete, 2011), crown plasticity (Seidel, Leuschner, Müller, & Krause, 2011), crown competition (Metz et al., 2013), and CVs (Fernández‐Sarría et al., 2013; Metz et al., 2013). Often, α‐shape and voxel grid approaches are used in combination (Fernández‐Sarría et al., 2013; Metz et al., 2013; Seidel et al., 2011), mainly because they enable faster point cloud processing and simplification.

So far, many α‐shape and voxel‐based approaches that describe crown metrics have been developed with one‐time data sets. In ecological studies, however, the temporal dynamics of forests and tree communities are of particular importance. Studies with multitemporal point cloud data from forests are rare and mainly focused on estimating changes in diameter at breast height or aboveground biomass of individual trees (Liang, Hyyppä, Kaartinen, Holopainen, & Melkas, 2012; Srinivasan, Popescu, Eriksson, Sheridan, & Ku, 2014). In addition, some studies used noninteracting trees (no crown overlap) and no real forest examples (e.g., Fernández‐Sarría et al., 2013; Moorthy et al., 2011). However, the interaction of tree crowns and the temporal development and outcome of this competition for canopy space are of particular interest in forest ecology (Pretzsch, 2014). Therefore, methods that can be applied repeatedly in complex forest environments to measure tree crown interactions are desired. In addition, up to now, no studies exist that analyze the spatiotemporal dynamics of crown space occupation in dense mixed‐species forests.

The main objective of this study was to introduce a novel method to analyze spatiotemporal dynamics in canopy occupancy at the individual tree and local neighborhood level using high‐resolution 3D TLS data. Specifically, we aim to analyze the canopy occupancy at tree level by identifying and quantifying the crown space and volume occupied by the respective tree only or shared with neighboring trees (i.e., crown intersection volume). Furthermore, at the local neighborhood or plot level, we intend to quantify the extent of canopy space occupation and complementary space use. Finally, our goal was to precisely detect and quantify the changes in canopy occupancy through time. We present an exemplary application of our method based on multitemporal TLS data from a large‐scale biodiversity‐ecosystem functioning (BEF) experiment in subtropical China. Annual scan data (2012–2016) are available for 30 experimental plots comprising 1258 trees. Here, we processed a subset of four plots with a total of 113 tree individuals and conducted change detection analyses for the census interval 2014–2015.

## 2 MATERIAL AND METHODS

### 2.1 TLS data acquisition

TLS data were acquired from one BEF‐China experimental site. The site is located in a hilly subtropical region near Xingangshan Township, Jiangxi Province, China (29° 08′‐11′ N, 117° 90′‐93′ E, altitude 105–275 m a.s.l.). Mean annual temperature is 16.7 °C, and mean annual precipitation is 1,821 mm. Each of the 556 plots in the BEF‐China experiment has a projected area of 667 m^2^ (25.8 × 25.8 m) and was planted with 400 (20 × 20) trees with a horizontal planting distance of 1.29 m in 2009 and 2010. The plots have species richness levels of 1, 2, 4, 8, 16, and 24 (for a detailed description see Bruelheide et al., 2014).

Our analyses focused on two deciduous tree species: *Castanea henryi* (Skan) Rehd. et Wils. (Fagaceae) and *Nyssa sinensis* Oliver (Nyssaceae). We selected two monospecific plots (one of each species) and two mixed‐species plots, where only trees of these two species were planted. The TLS campaigns focused on trees within the core area of each plot, consisting of the central 6 × 6 = 36 planting positions (Supporting Information Figure S1). These trees were also dendrometrically measured by conventional methods in annual field inventories carried out at the end of the main growing season (September–October).

Two TLS scan campaigns were conducted under leaf‐off conditions in March 2014 and March 2015. A standardized multiple‐scan setup, comprising nine scan positions, was established for all plots in both years (Supporting Information Figure S1). To coregister the point clouds from multiple scans, several highly reflective standardized reference targets (polystyrene spheres and checkerboards) were placed within and around the core area.

All scans were performed with a phase‐shift FARO LS Focus 3D (FARO Technologies Inc., FL, USA). Panorama scans with a maximal field view of 305 × 360° (vertical × horizontal) were conducted with a vertical and horizontal angular step size of 0.036° resulting in spatial resolutions of 1.3, 3.1, and 6.3 mm at distances of 2, 5, and 10 m, respectively.

In 2015, mean height and stem diameter at ground height of the studied trees ranged from 4.3 to 7.5 m and 5.1 to 8.6 cm, respectively (Supporting Information Table S1). The number of trees that were identified and extracted from the TLS data in the two study years was smaller than 36 per plot because (a) not all trees planted in 2009 and 2010 survived until 2014 (and one tree died between 2014 and 2015) and (b) as also found by Li et al. (2014), trees <40 cm height could not be detected by the TLS setup used in this study. In total, 113 and 112 trees were processed and analyzed in 2014 and 2015, respectively.

### 2.2 TLS data preprocessing

Our presented method uses multitemporal data of individual trees. Therefore, to effectively apply our modeling approach we preprocessed the TLS data in the following way.

#### 2.2.1 Coregistration of multiple scans

For each plot, all scans were coregistered within FARO SCENE software (FARO Technologies Inc., version 5.2, 2014). Before registration we applied a stray point filtering and a minimum intensity filter (with the standard FARO SCENE settings) to remove stray and noise points. The coregistration of the nine scans per plot was performed automatically, based on the reference spheres and checkerboard targets. The relative registration accuracies for the single scans in each plot were all within 3 mm.

#### 2.2.2 Extraction of tree individuals and terrain model

From the coregistered point clouds, all identified tree individuals were manually extracted as separate individual tree point clouds in CloudCompare software (CloudCompare, version 2.6.1, 2015). Point cloud coordinates and intensity values were stored in an ASCII format in a local Cartesian coordinate system for each plot. Digital terrain models (DTM) were automatically extracted from the coregistered plot point clouds using a raster grid size of 5 cm that extracted the lowest point in each raster cell. Empty grid cells were filled with the interpolated height of neighboring cells (Akima & Gebhardt, 2015).

#### 2.2.3 Single tree point cloud filtering and subsampling

In case of remaining noise points, we additionally applied a statistical outlier removal filter (*n* = 10, σ = 1.5; Rusu & Cousins, 2011) on the individual tree point clouds. To achieve faster processing and reduction in point cloud size, each point cloud was spatially subsampled, resulting in minimum distances between two neighboring points of 1 cm. This decreased the total number of points on average by about 97% while retaining a sufficient amount of structural information for the subsequent α‐shape and voxel grid modeling. Note that, the spatial subsampling is an optional step, mainly for computational speed‐up, as our method may also be used with full resolution.

#### 2.2.4 Multitemporal coregistration

In our case, the coregistered point clouds from each plot had a unique local coordinate system. This is explained by missing survey markers in our tree diversity experiment. For other field studies, such markers may exist and multitemporal coregistration may be achieved more easily. Nonetheless, we were able to accurately coregister our point clouds from subsequent years. Here, we used the tree positions at ground level as an independent reference framework for the multitemporal coregistration (Supporting Information Figure S2).

Each tree position was derived automatically from the extracted and filtered tree point cloud (before the spatial subsampling). To extract the tree position, a circle fit was performed using a slice of the stem points at a height of 5 cm above ground. The resulting circle center then marked the tree position (Bienert, Scheller, Keane, Mullooly, & Mohan, 2006). Tree positions from the year 2014 were used as reference. Subsequently, the individual trees and DTM from the following year were coregistered using a 2D conformal transformation in a least squares fashion (Ghilani & Wolf, 2010). The scanner and the respective point clouds were assumed to be levelled and not rotated with respect to the horizontal level. In order to align the trees to the same height level, we projected the tree positions onto the respective DTM and calculated a mean height level for each plot and year. A height correction was then applied to the point clouds to achieve a match (Supporting Information Figure S3). All multitemporal coregistrations showed a mean distance between all matched tree positions of 1.8 cm ± 1.0 cm *SD*.

### 2.3 Voxel grid tree and plot modeling

For the individual tree crown and canopy occupancy analyses, we used a voxel grid approach. The term voxel grid defines the commonly used 3D spatial reference framework of unique voxels. A voxel represents the smallest cubic unit in the grid with a defined edge length and unique position. The voxel size was orientated on the size and dimensions of the nonexistent leaves during the scan campaign. Besides the stem and branches, the voxels should be large enough to model the spatial expansion and occupations of the species‐specific leaves. Here, we used a voxel size of 10 cm (i.e., voxel volume of 0.001 m^3^). Our canopy occupancy analysis consists of two major steps: (a) individual tree modeling and (b) plot modeling.

In the first step of our analysis, we separated each tree into two parts, the stem and the crown. As crown, we defined all points above the crown base height (CBH), which was defined as the height of the bifurcation point of the lowest live branch of the tree. CBH data were obtained from conventional field inventories because living and dead branches are difficult to differentiate directly from the TLS data. Points below the CBH were defined as stem point. The two resulting compartments, stem and crown, were subsequently processed individually (see also Supporting Information Figure S4).

**Figure 1:**
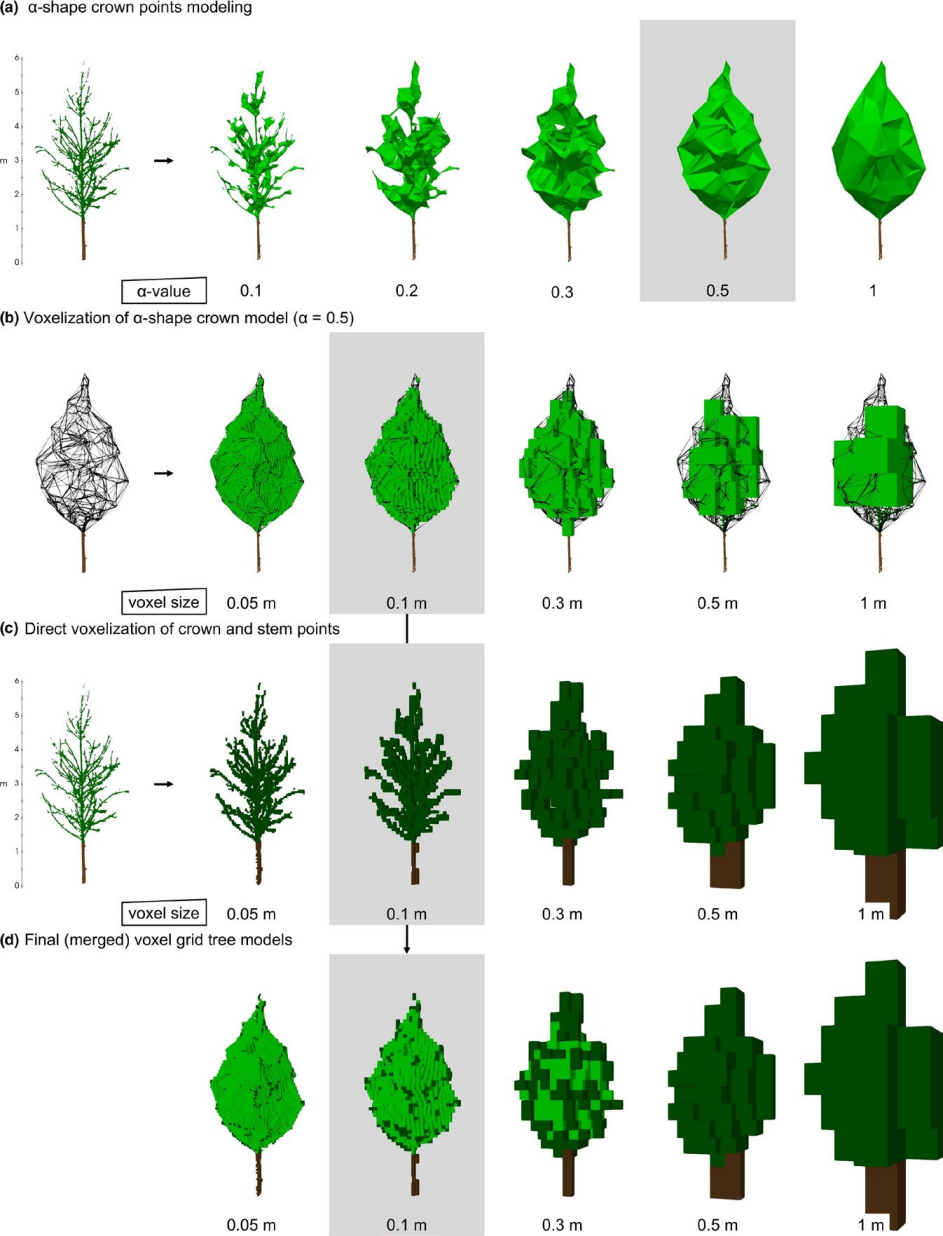
Steps in voxel grid tree modeling. Each step is processed for five different values of the model parameter α‐value or voxel size. Gray highlighted models match the model parameter values of our analyses: (a) α‐shape crown models (lightgreen). (b) α‐shape crown model voxelization (lightgreen). (c) Direct voxelization of the crown (darkgreen) and stem (brown) points. (d) Completed merged voxel grid tree model

For crown space modeling, we defined two representations of the crown. The first representation of the crown was formed by a 3D α‐shape (Edelsbrunner & Mücke, 1994) around the crown points (Figure 1a). Here, we used the value α = 0.5. The enclosed crown space of the α‐shape was then converted into a voxel grid (Figure 1b).

The second representation was a direct voxelization of the crown and stem points, that is, the branches as well as the stem points (Figure 1c). Both representations were then merged together into one crown model (Figure 1d).

**Figure 2:**
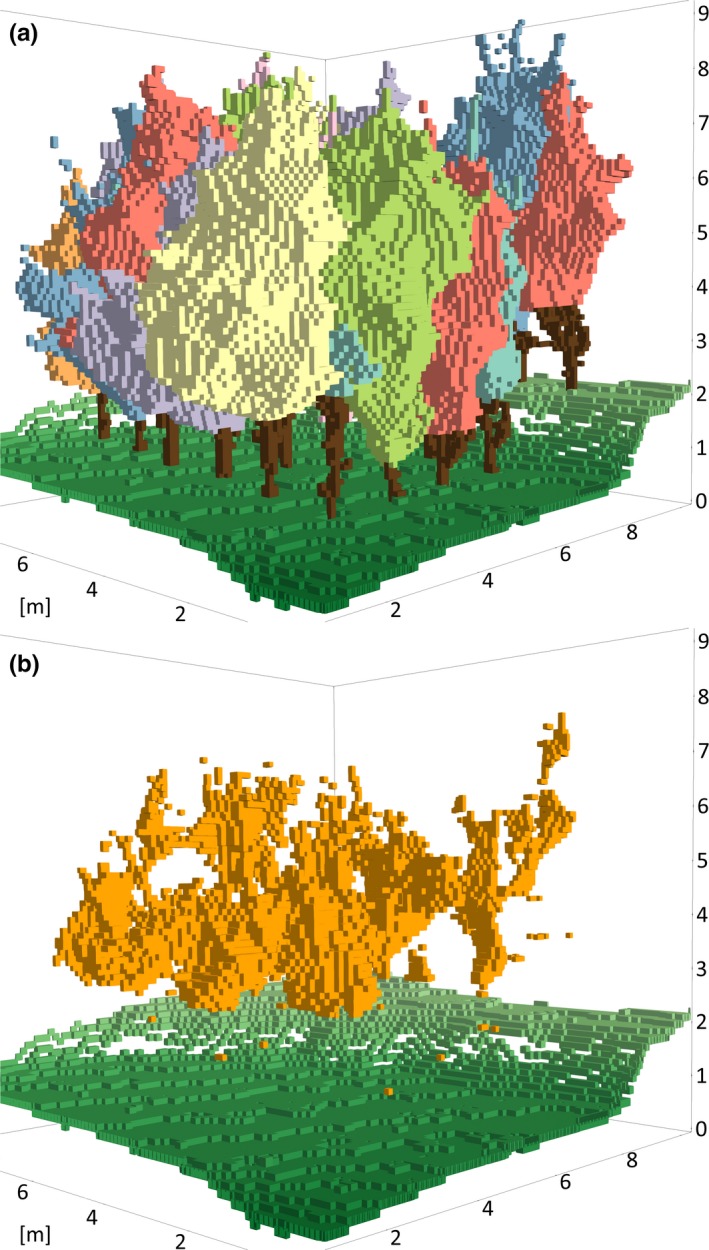
Voxel grid tree modeling at plot level (plot E34, 2014, voxel size = 10 cm). (a) Single tree voxel grid models. (b) All voxels occupied by two trees (orange). Empty voxels are not apparent. Gaps between ground voxels (green) are caused by differences in elevation larger than the voxel size

**Figure 3:**
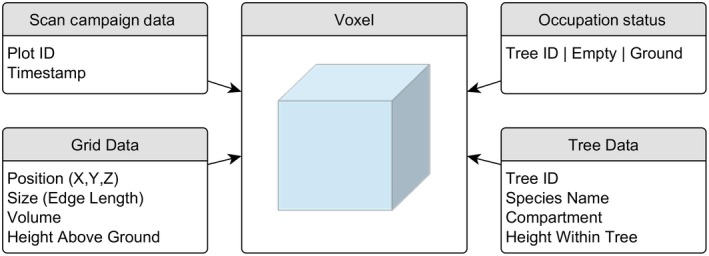
Voxel attributes within the voxel grid data table

In the second step of our method, we first created an empty voxel grid with the spatial extent defined by the DTM and the voxel grid tree models of the respective plot. The unique voxel positions (X, Y, Z) of the DTM allowed us to calculate the height above ground for each voxel within the grid (Height _Voxel (X,Y) _= Z _Voxel (X,Y)_ − Z _DTM (X,Y)_). All voxel grid plot models had the same maximum height above ground of 10.3 m. We choose this height as it corresponded to the maximum height above ground of all tree models in the data set. In the next step, each tree model was added to the empty plot model (Figure 2a). Spatial canopy occupations in the voxel grid are defined by each voxel of a tree (assigned to crown or stem). These voxel were mapped to the plot by the voxel's unique position (X, Y, and Z) as the joint index variable. If two or more trees were assigned to the same voxel position, each voxel extended the voxel grid data table by a new record. Canopy space shared by two or more trees could be identified by the same unique voxel position (Figure 2b). Figure 3 gives a structured overview of the main attributes that were attached to each voxel record in the generated voxel grid data table on plot level.

### 2.4 Analyses of canopy occupancy in voxel grids

Changes of canopy space occupancy were carried out for the 4 × 4 inner core target trees only (Supporting Information Figure S1). These are the trees for which we had full information on all neighbors. The total CV was calculated by the summation of the tree's crown voxels. Voxel volumes defined by overlapping crowns were summed up as the crown intersection volume (CIV). We distinguished between a single and multiple (two‐, three‐, or fourfold) occupation of voxels.

At plot level, we calculated the absolute voxel volumes for each type of occupation for a specific canopy space (investigation area). This investigation area was defined by a ground area of 6 m × 6 m (centered against the tree positions of the inner core trees) and a height of 10.3 m above ground, resulting in a total space volume of 370.8 m^3^ (Supporting Information Figure S5). This height was derived from the maximum height above ground of all occupied voxels in all investigation areas.

Changes in occupation were calculated by the differences between the 2 years. Hotspots and patterns in canopy occupancy were identified as areas with high levels of occupation and the rates of change. For each plot, we aggregated the voxel grid data for two different strata (canopy layers), each of 1 m in height. Within these layers, the total number of occupations and change rates was summed up. Occupations were categorized as empty, single, and multiple, and changes in occupation as increased, decreased, and unchanged. Figure 8 shows a visualization of the categories, that is, a heat map in the voxel grid. The voxel color represents the type of occupation and changes in occupation (Wilkinson & Friendly, 2009).

All analyses were performed using R (R Core Team 2016); point cloud voxelizations and voxel grid generations were carried out by specially developed functions. α‐shapes were generated and converted into the voxel grid using the R‐package alphashape3d (Lafarge & Pateiro‐Lopez, 2016).

## 3 RESULTS

### 3.1 Canopy occupancy at the individual tree level

**Figure 4:**
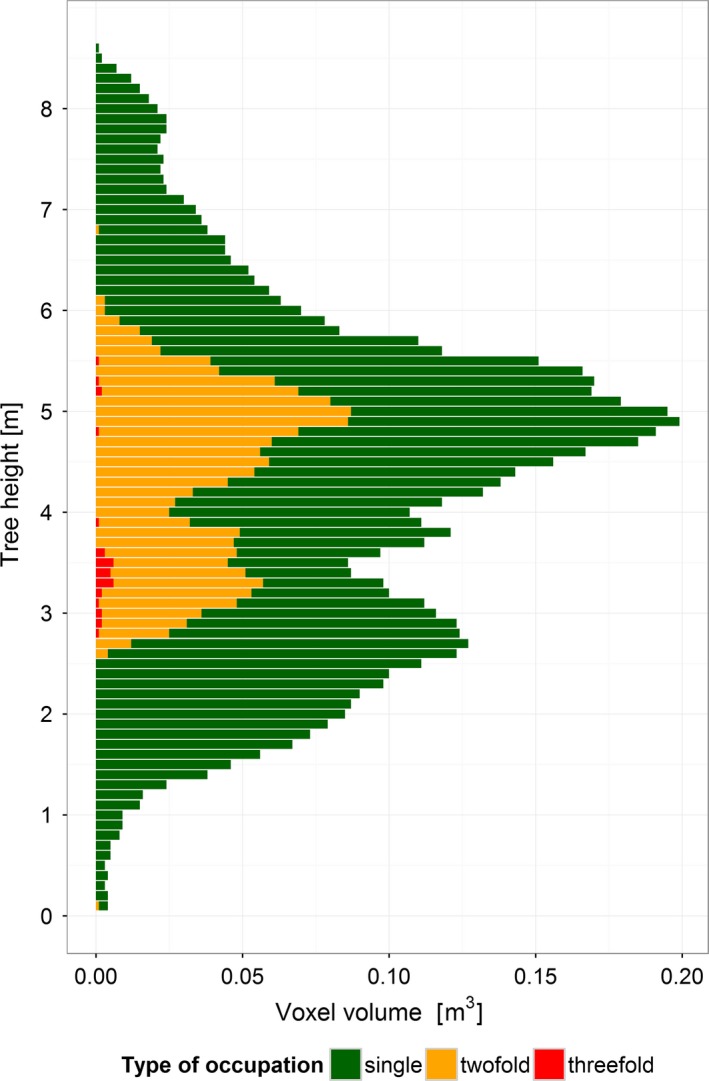
Variation in occupied voxel volumes with tree height for an individual of *Castanea henryi* (plot E34, 2015). Each bar represents a 10‐cm‐height interval

Canopy occupancy strongly varied with tree height (Figure 4). For example, at the level of a single crown of *C. henryi*, the maximum total volume per voxel layer (0.2 m^3^) was found at a height of 4.9 m. The majority of CV was occupied exclusively by this tree (76.4%), whereas 23.1% and 0.5% were occupied twofold and threefold, respectively (i.e., by one and two neighboring trees).

**Figure 5:**
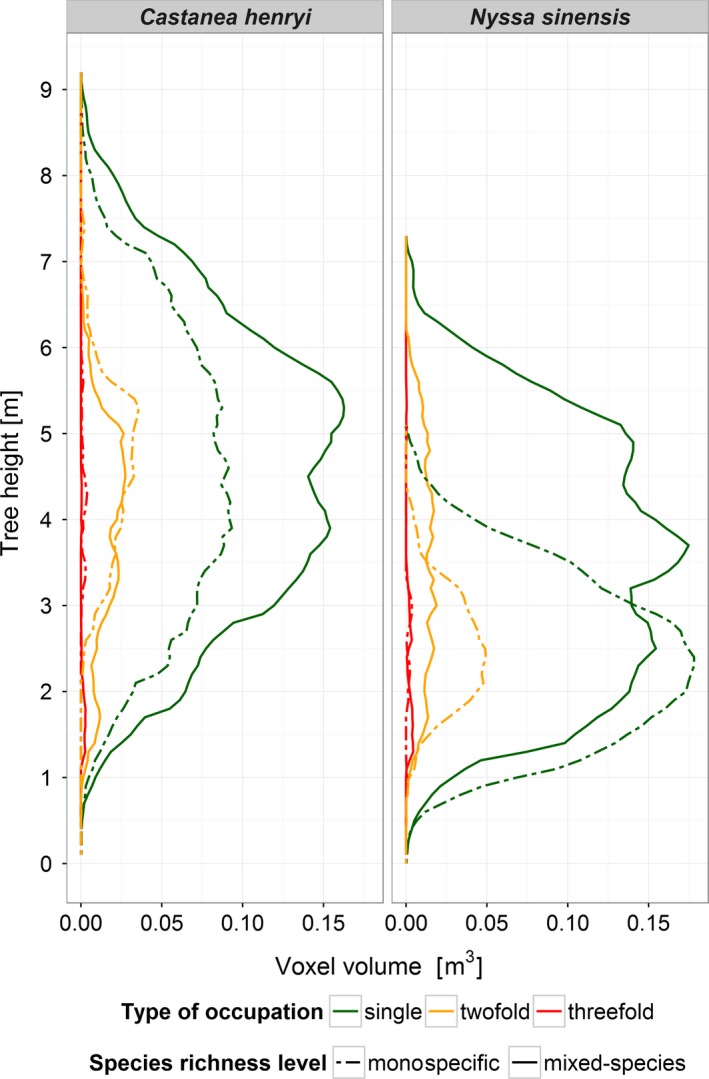
Differences in mean crown voxel volumes across tree height between species and occupation type for monocultures and two‐species mixtures. Data represent voxel volumes of all inner core trees scanned in 2015

Similarly, we found height dependency of crown occupation when considering all inner core trees (Figure 5). For monocultures, mean CV followed a normal distribution, but the maximum volume per voxel layer was located at a greater tree height in *C. henryi* than in *N. sinensis*. In the mixed‐species plots, however, the vertical distribution strongly deviates from a normal distribution. The vertical distributions of twofold occupation in monocultures largely followed those of single occupation (Figure 5), whereas the mixed‐species plots showed a tendency toward a more even vertical distribution.

**Figure 6:**
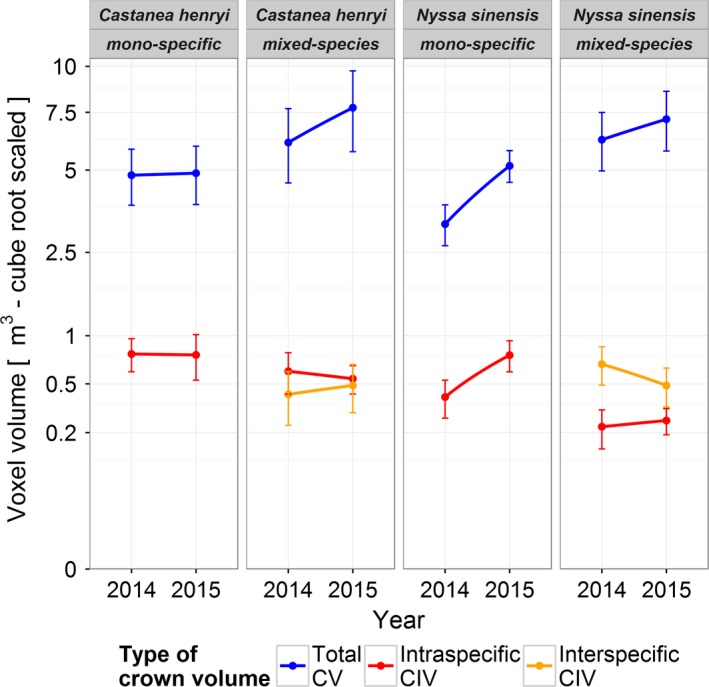
Mean (± 1 *SE*) total crown volume (CV) and crown intersection volume (CIV) for the inner core trees with all direct neighbors grouped by tree species, species richness level, and year. Intraspecific (red) and interspecific (orange) CIVs are shown. *Y*‐axis is cube root scaled for easier comparison between smaller CIV and larger CV values

In all plots, intraspecific CIV decreased over time in one species (*C. henryi*, mono: −0.01 m^3^; mixed: −0.07 m^3^), but increased in the other (*N. sinensis*, mono: +0.34 m^3^; mixed: +0.03 m^3^; Figure 6). Interspecific CIV showed the opposite pattern (*C. henryi*: +0.07 m^3^; *N. sinensis*: −0.19 m^3^) (Figure 6).

### 3.2 Canopy occupancy at plot level

**Figure 7:**
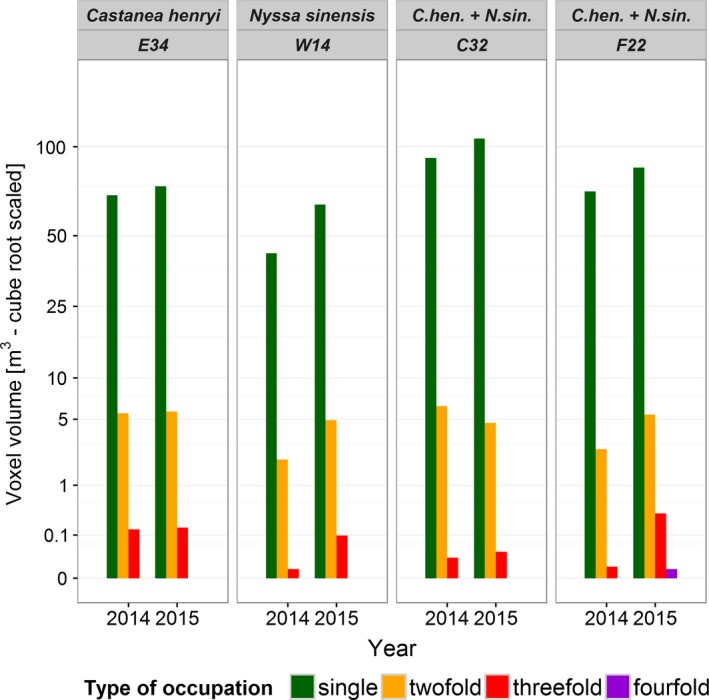
Absolute crown voxel volumes conditioned on occupation type, plot, and year. *Y*‐axis is cube root scaled for easier comparison between smaller multiple and larger single occupations

In the monocultures, canopy occupancy was higher in C. *henryi* than in *N. sinensis* (Figure 7). In mixed‐species plots, the occupied space volumes were generally higher than in monocultures. Twofold and threefold occupation were found in all plots, fourfold occupation occurred only in one mixed‐species plot in 2015.

### 3.3 Change detection in canopy layers

**Figure 8:**
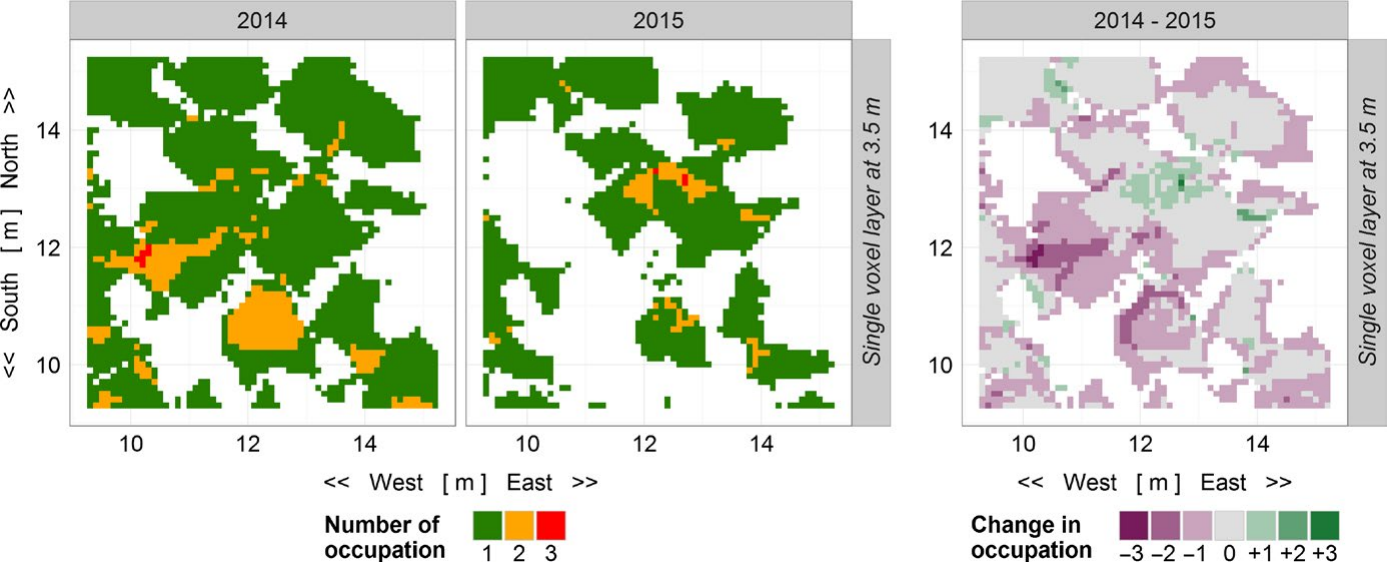
Heat maps of the monospecific plot of *Castanea henryi* (plot E34) within the lateral dimensions of the investigation area (6 m × 6 m). Left panels: voxel occupations at 3.5 m above ground in 2014 and 2015. Right panel: calculated changes in voxel occupation between both years

Using the monospecific plot of *C. henryi* (E34) as an example, the single voxel layer at 3.4 m above ground revealed a high turnover in voxel occupations within 1 year (Figure 8). Overall, the amount of empty canopy space volume increased and the number of voxels occupied by two or more individuals decreased. One new hotspot of threefold occupation emerged in 2015.

**Figure 9:**
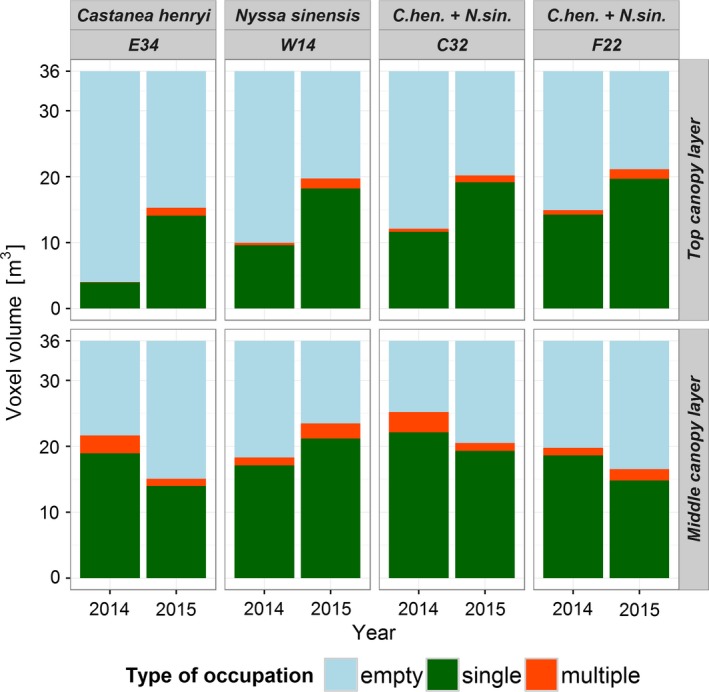
Crown voxel volumes conditioned on plot, year, canopy layer, and occupation type. Canopy layers are defined by a ground parallel segment of 1 m height within an investigation area of 6 m × 6 m (total volume of 36 m^3^). Top canopy layers are centered on the plot's mean tree height in 2014 and middle canopy layers on mean crown center height of the plot in 2014

**Figure 10:**
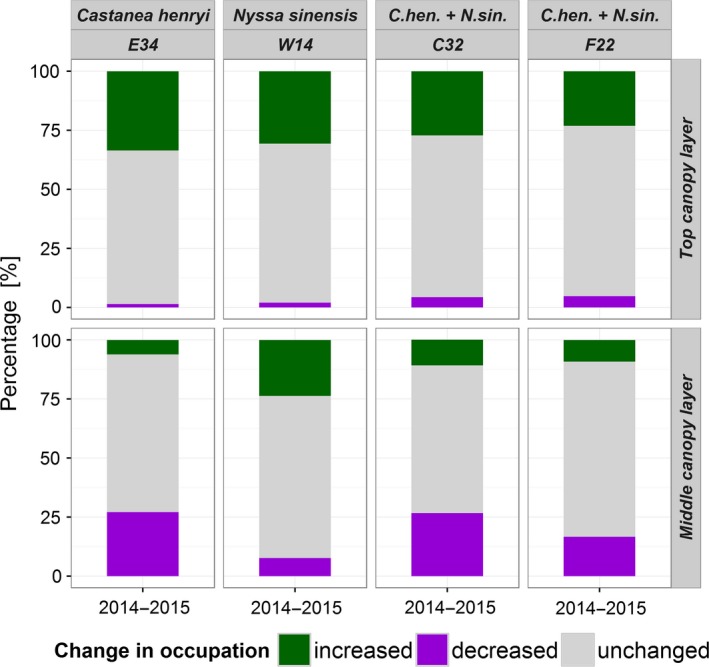
Changes in voxel occupation between 2014 and 2015, shown as percentages of voxels with an increase (green) or decrease (violet) in occupation level or those which remained unchanged (gray)

For all plots, the top layers showed a clear trend in increasing canopy occupancy (Figure 9). This translates to a higher proportion of increasing than decreasing occupations for the top canopy layers (Figure 10). Changes in the middle canopy layer showed the opposite pattern (Figure 9). The highest decrease in single and multiple occupations was found for plots E34 (−4.96 m^3^) and C32 (−1.83 m^3^), respectively. Consequently, a lower proportion of increasing than decreasing occupations can be found for the middle canopy layers (except plot W14; Figure 10). A majority of all voxels showed no change in occupation over time (65%–72% in the top canopy layer and 63%–74% in the middle canopy layer; Figure 10).

## 4 DISCUSSION

We present the first description of a voxel grid‐based method to directly and nondestructively analyze the canopy occupancy at the individual tree and local neighborhood level. This highly precise and reproducible method employs high‐resolution 3D point clouds obtained by TLS. In forests, the interactions of tree crowns and the outcome of these interactions for canopy space filling are of high importance (Pretzsch, 2014). Any method that could advance our understanding of these dynamics has to be applied in complex forest environments. However, studies analyzing crown characteristics of individual trees often used noninteracting trees (no crown overlap) or examples from outside forests (e.g., Fernández‐Sarría et al., 2013; Moorthy et al., 2011). Furthermore, not only the spatial constellation of neighboring trees but also the temporal dynamics of individual tree crown structure and canopy space filling are important aspects in forest research. However, such studies with multitemporal point cloud data analyses are rare, mainly focusing on standard tree dendrometrics or wood volume estimations (Liang et al., 2012; Srinivasan et al., 2014). Our new method is, thus, a very promising approach which should lead to important advances in several aspects of forest ecological research, such as investigations on crown growth, morphology and plasticity (Longuetaud, Piboule, Wernsdörfer, & Collet, 2013; Schröter, Härdtle, & von Oheimb, 2012), canopy structures and canopy packing (Jucker, Bouriaud, Coomes, & Baltzer, 2015; Morin, 2015; Pretzsch, 2014), crown‐related tree interactions and competition (Fichtner, Sturm, Rickert, von Oheimb, & Härdtle, 2013; Lang et al., 2012; Potvin & Dutilleul, 2009; Thorpe, Astrup, Trowbridge, & Coates, 2010), and niche differentiation and spatial complementarity (Ishii & Asano, 2010; Sapijanskas, Paquette, Potvin, Kunert, & Loreau, 2014; Williams, Paquette, Cavender‐Bares, Messier, & Reich, 2017), including changes over time.

By making use of the TLS data for the crown modeling, there is no need to take a geometric shape as a basis (such as cone, paraboloid, and hemisphere; Fernández‐Sarría et al., 2013). To the contrary, the strong crown plasticity of individual trees becomes apparent (Figure 4). In comparison with the respective monocultures, the two study species showed different patterns of vertical CV distribution in the mixed‐species plots (two peaks in *C. henryi* and three peaks in *N. sinensis*; Figure 5), which can be seen as an interspecific complementary in canopy space use. Recently, Williams et al. (2017) showed in a tree biodiversity experiment that physical niche partitioning in canopy space, induced by both interspecific differences in crown architecture and neighborhood‐driven crown plasticity, may be a key factor driving positive relationships between tree diversity and biomass production in forests. This study used relatively rough one‐time estimates of the outside dimensions of the tree crowns in very young trees (4 years after planting). With our approach it will be logistically feasible also to study older trees of several meters in height (Figure 5), to add the within‐crown component of tree–tree interactions (CIV and type of occupation), which has never been considered so far in canopy space filling analyses, and to elucidate the important temporal dynamics.

The accuracy of tree parameter derivation from TLS data depends on the original quality of the point clouds, which is affected by (a) the resolution and number of scans, (b) the tree stand density, structural complexity and dimensions of individual trees affecting the amount of occlusions, and (c) the movement of tree compartments due to wind. Shadowing constitutes a major challenge for point cloud assessments. The voxel grid approach, however, compensates for and is less susceptible to the inaccuracies and potential limitations in TLS data quality, enabling highly accurate crown modeling.

The voxel grid models of individual tree crowns represented the first important outcome of our method. Based on these models, we were able to precisely model individual crown structures and estimate their volumes, which is confirmed when comparing a set of results of our TLS data analyses with those from classical dendrometry volume calculation (Supporting Information Figure S6). In addition to these classical estimation methods, point cloud analyses enable covering the full asymmetric shape of the crowns in high resolution. A further significant advantage is that we were able to assign all CIVs to intraspecific and interspecific tree interactions.

The second important outcome of our approach is the voxel grid model on plot level. This model combines the individual tree models and the DTM. The generated voxel grid data table is very robust and user‐friendly even in the case of large amounts of data. For standard forest inventories, both circular and square plot areas could be defined. Besides the canopy layers as exemplified in this study, more complex polygonal boundaries or search areas like cones (Metz et al., 2013) could be established as investigation areas. Because the plot model considers empty voxels from the beginning, it can also be used to analyze gap fractions within forest stands.

A number of points need to be taken into account at the individual tree level to further improve crown modeling. As we scanned the trees under leaf‐off conditions, we were able to manually extract single trees and determine interleaved branches and overlapped crowns with high resolution. However, scanning trees without leaves do not represent the full extent of the crown's dimension. Leaves will occupy additional space around the branches. In general, voxel sizes from 10 to 30 cm seemed to be most adequate to represent the spatial occupations of the missing leaves while preserving asymmetric crown shapes. This is mainly relevant at the surface of the crown's shape and when leaves are large or feathered. α‐shapes primarily model the unoccupied but shaded space inside the crown. In combination with the direct point cloud voxelization, we suggest α‐values between 0.3 and 1.0. Compared to the step of α‐shape voxelization, where the spatial expansion of the voxel grid model is limited by the α‐shape (Figure 1b), the direct voxelization of the crown and stem points is more sensitive to changes in voxel size. For small trees, a voxel size larger than 0.5 m will lead to an overexpansion of the voxel grid crown model (Figure 1d) and overestimation of CVs and CIVs.

The presented method is flexible enough to allow more attributes than the presented ones to be assigned to the voxel grid data table (Figure 3). Based on our TLS data, we could determine whether a voxel was occupied by a branch or was part of the crown's surface area or inner crown space. This might help to address and better understand the origins of changes in occupation, for example, caused by mechanical abrasion or competition for light (Hajek, Seidel, & Leuschner, 2015). Even geographical coordinates can be added to model specific light absorption. Furthermore, a differentiation of branch order levels can be performed using both voxel‐ and cylinder‐based tree modeling approaches (Gorte & Winterhalder, 2004; Hackenberg et al., 2014; Raumonen et al., 2013). The estimation of leaf area densities of trees scanned under leaf‐on conditions (in particular using full‐waveform scanners) can also be used to further describe and differentiate the voxel's potential degree of shadowing (Hosoi & Omasa, 2006).

Despite of the advantages of TLS data analysis when using our method, some challenges remain when managing a large sample size such as that used by Jucker et al. (2015). Nowadays, many TLS data processing steps are straightforward. The coregistration of multiple scans comes with a fully automated workflow, in some cases even without the need for additional reference targets. The latter makes data acquisition in the field even less time‐consuming. Currently, the most important challenge is the lack of methods for fully automatic and reliable individual tree segmentation from unorganized point cloud data. This step in data processing is still a bottleneck, which results in a large amount of manual and subjective point cloud data manipulation. The analysis of tree interaction which considers overlapping tree crowns requires the identification and assignment of crossing branches (trees scanned under leaf‐off condition) or the individual crown surface itself (leaf‐on condition). At present, only a subjective and manual top‐down segmentation process for each individual tree provides an immediate quality control of the segmented point cloud of each individual tree. It is, therefore, important to develop methods that come with largely automated data processing chains. In this context, it should be noted that TLS data sets are also an excellent documentation tool: Once new methods are available, the existing TLS data sets can easily be reanalyzed and all outcomes can be reassessed.

## CONFLICT OF INTEREST

None declared.

## AUTHOR CONTRIBUTIONS

CH initiated the approach and method. GvO, WH, and CH conceived the ideas and designed the research. CH and MK collected and compiled the data. CH analyzed the data and wrote the manuscript. All authors contributed critically to the drafts and gave final approval for publication.
