# Supplementary material for: A high‐resolution approach for the spatiotemporal analysis of forest canopy space using terrestrial laser scanning data
Source: Ecol Evol. 2018 Jun 11;8(13):6800–11. doi: 10.1002/ece3.4193 (PMC6053553; doi:10.1002/ece3.4193)
Supplement: Supplementary file 1 [file ECE3-8-6800-s001.docx]

# Supporting Information

**Tab. S1**: Total number of extracted trees per plot and species from TLS campaigns in 2014 and 2015. The species richness levels denote mono-specific (1) and two-species mixed (2) plots. Tree characteristics were obtained directly from the TLS data (TH = mean tree height, GD = mean stem diameter at ground).

| **Plot ID** | **E34** | **W14** | **C32** | **F22** |
| --- | --- | --- | --- | --- |
| **Species richness level** | **1** | | **2** | |
| **Year** | **2014 / 2015** | | | |
| ***Castanea henryi*** | **25 / 25** |  | **22 / 21** | **11 / 11** |
| TH [ m ] | 6.0 / 7.5 |  | 5.8 / 7.3 | 3.8 / 4.8 |
| GD [ cm ] | 6.2 / 7.2 |  | 6.4 / 8.2 | 4.1 / 5.1 |
| ***Nyssa sinensis*** |  | **27 / 27** | **10 / 10** | **18 / 18** |
| TH [ m ] |  | 3.6 / 4.3 | 5.6 / 6.2 | 4.7 / 5.8 |
| GD [ cm ] |  | 4.7 / 5.8 | 6.7 / 8.2 | 7.0 / 8.6 |
| **Total tree number** | **25 / 25** | **27 / 27** | **32 / 31** | **29 / 29** |


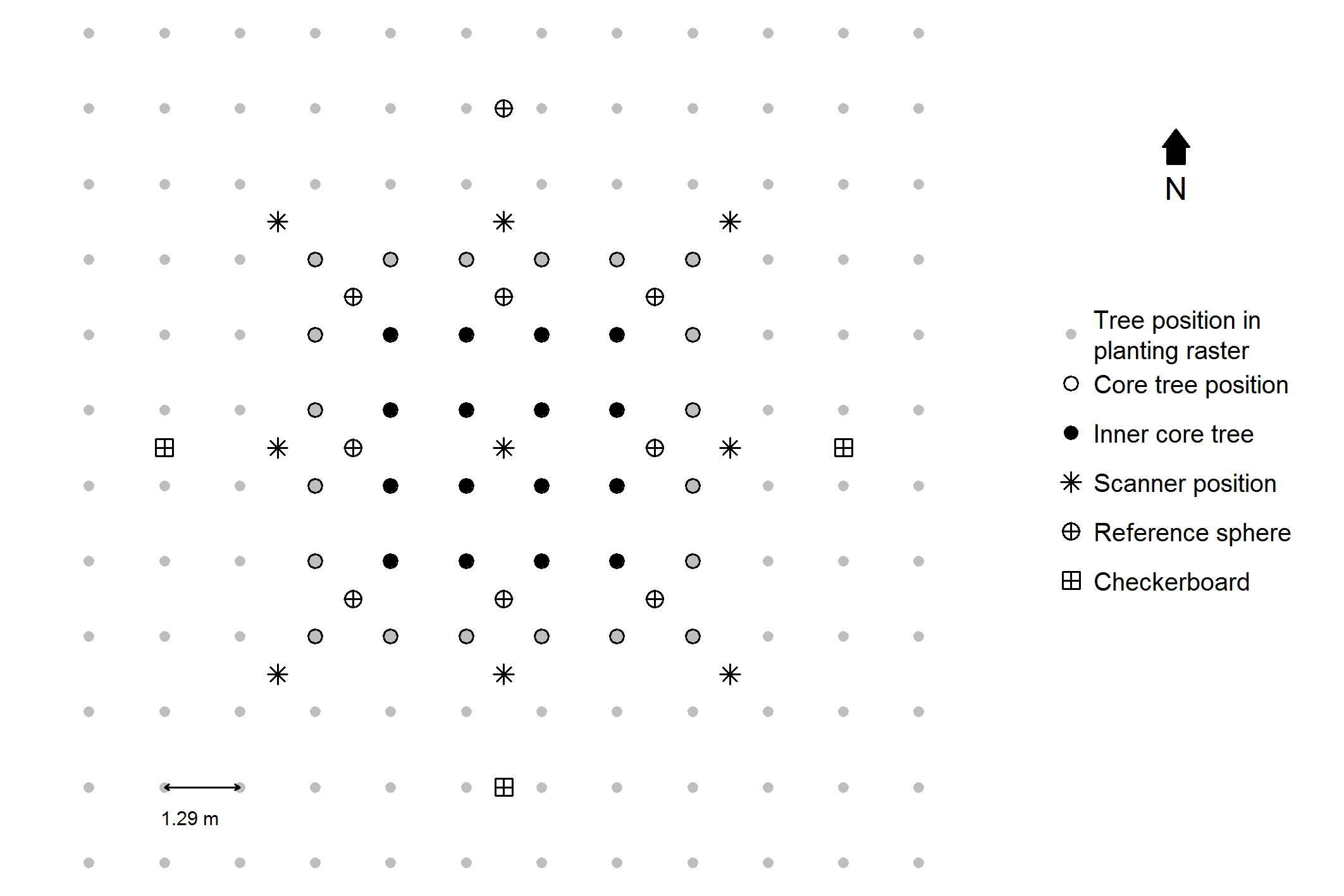


**Fig. S1**: Detail of the general plot layout showing the standardised multiple-scan setup to obtain TLS data for the core area (6 × 6 trees) in the experimental plots of BEF‑China. The 4 × 4 inner core trees represent the target trees.


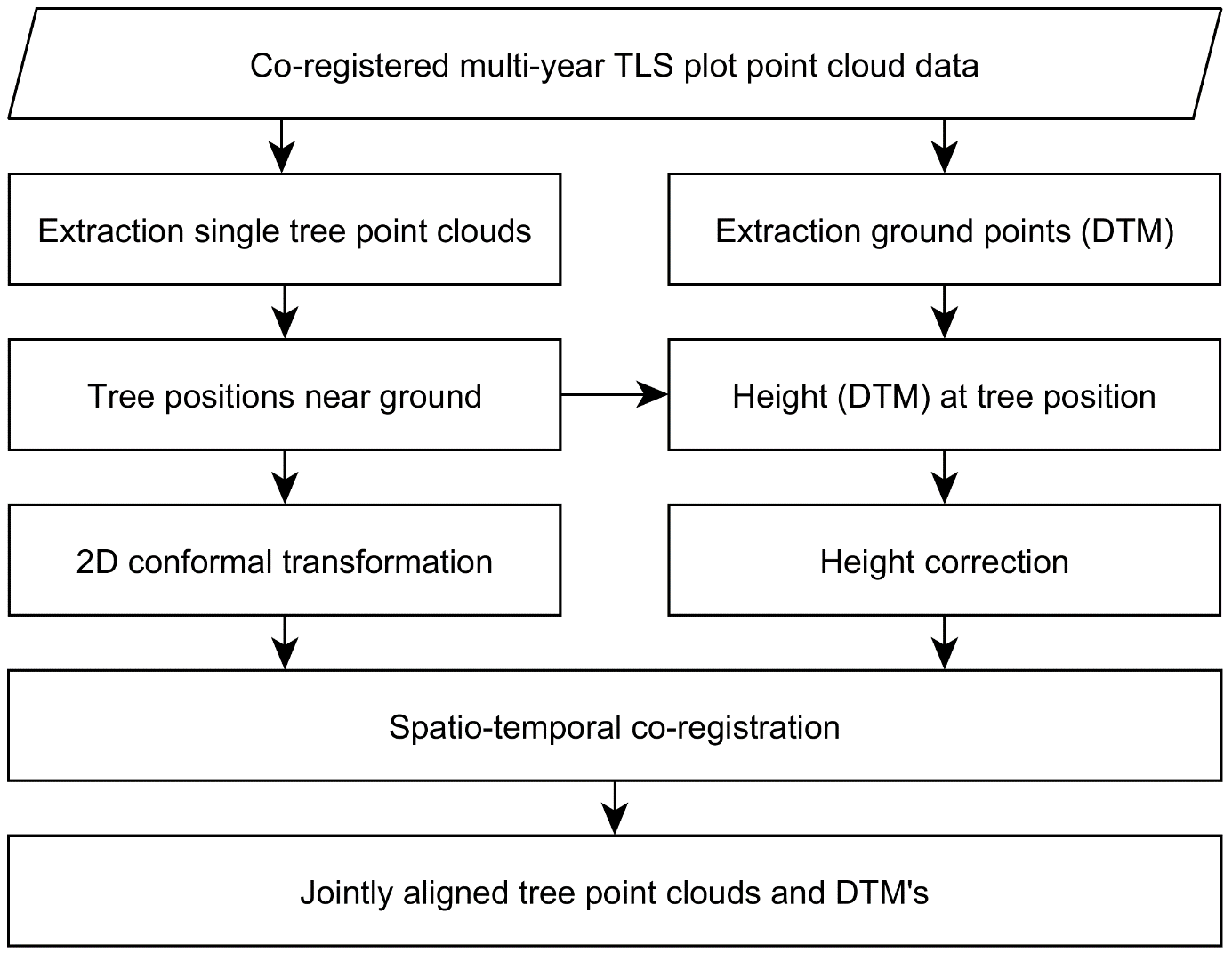


**Fig. S2**: Flow chart of the multi-temporal co-registration of repeated (e.g. annual) scanned plots.


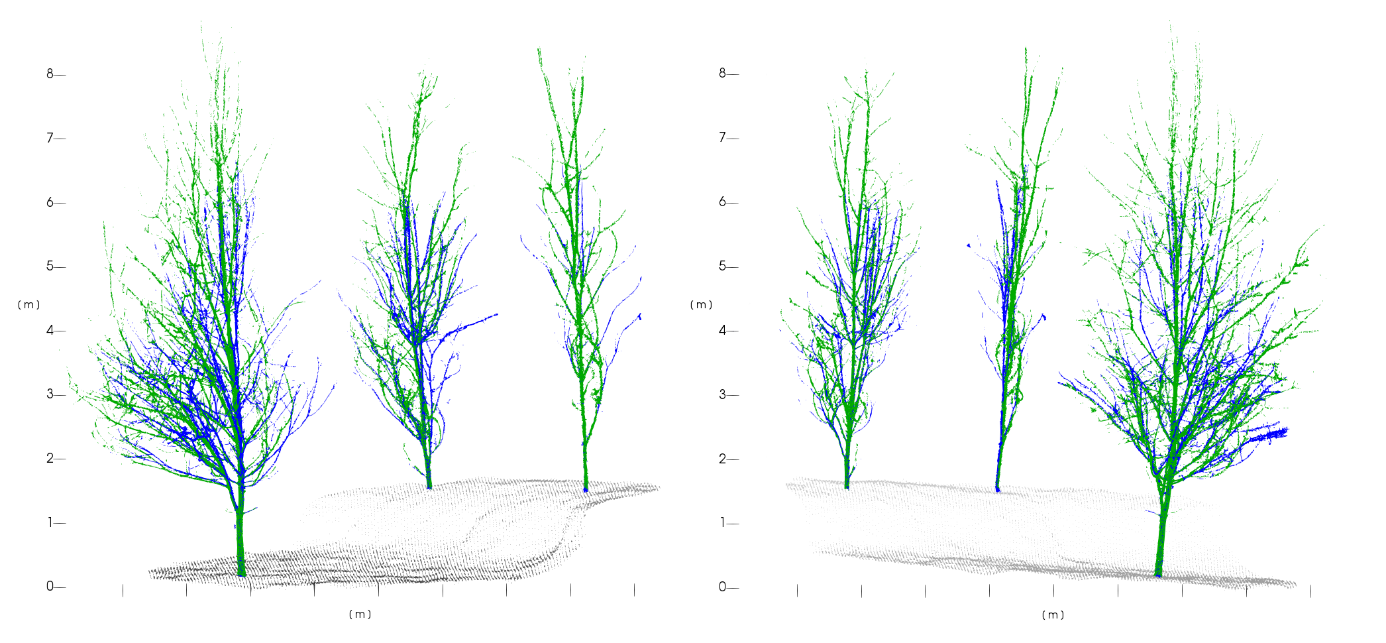


**Fig. S3**: Detail views (subset of three trees - left: heading north; right: heading east) of multi-temporal co-registered tree point cloud data for plot E34. Tree data from 2014 (blue) and 2015 (green) illustrate the high accuracy in alignment after the co-registration, originated in the tree positions on the ground.


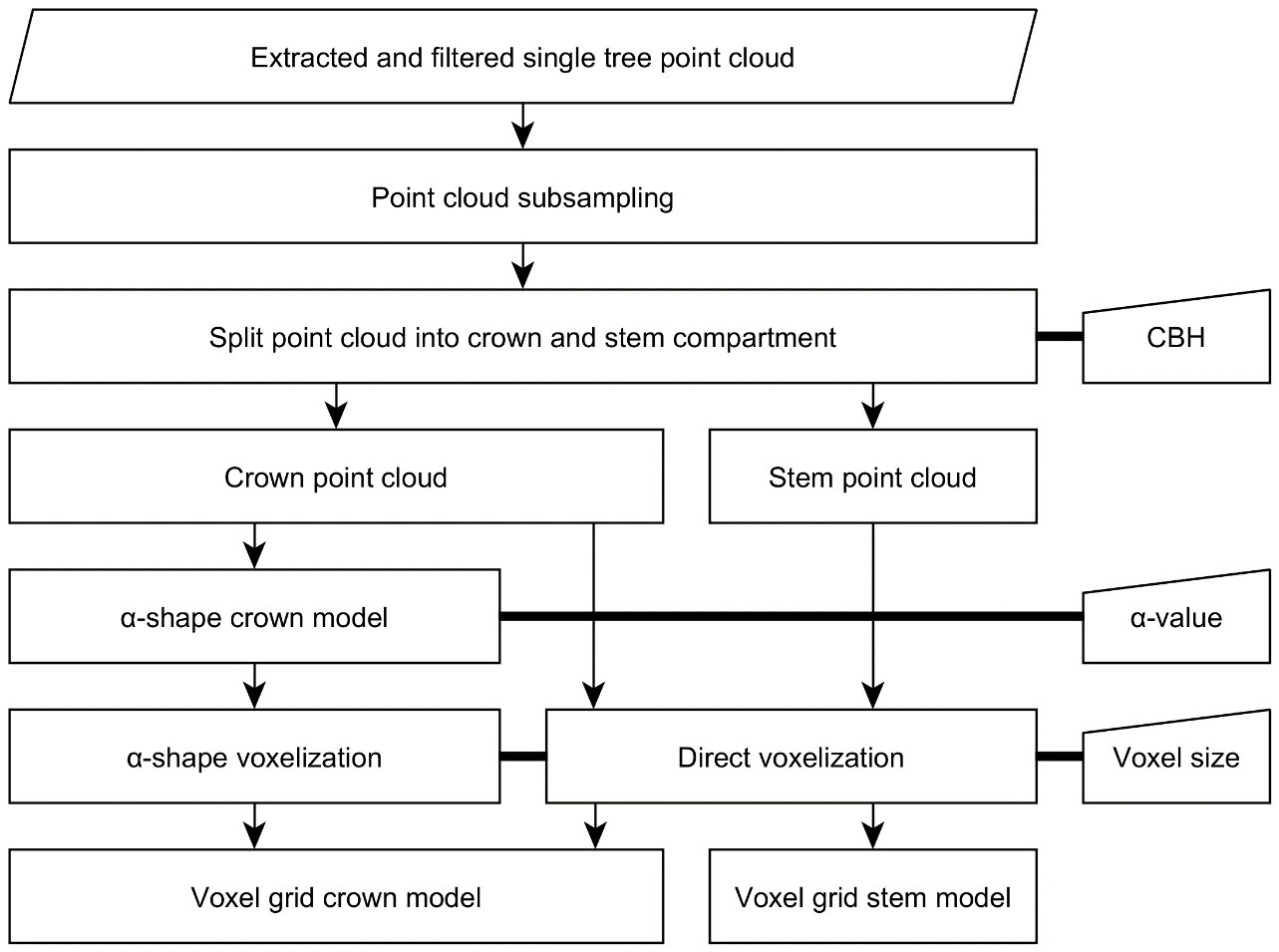


**Fig. S4**: Flow chart of the steps in voxel grid tree modelling including the α-shape crown modelling.


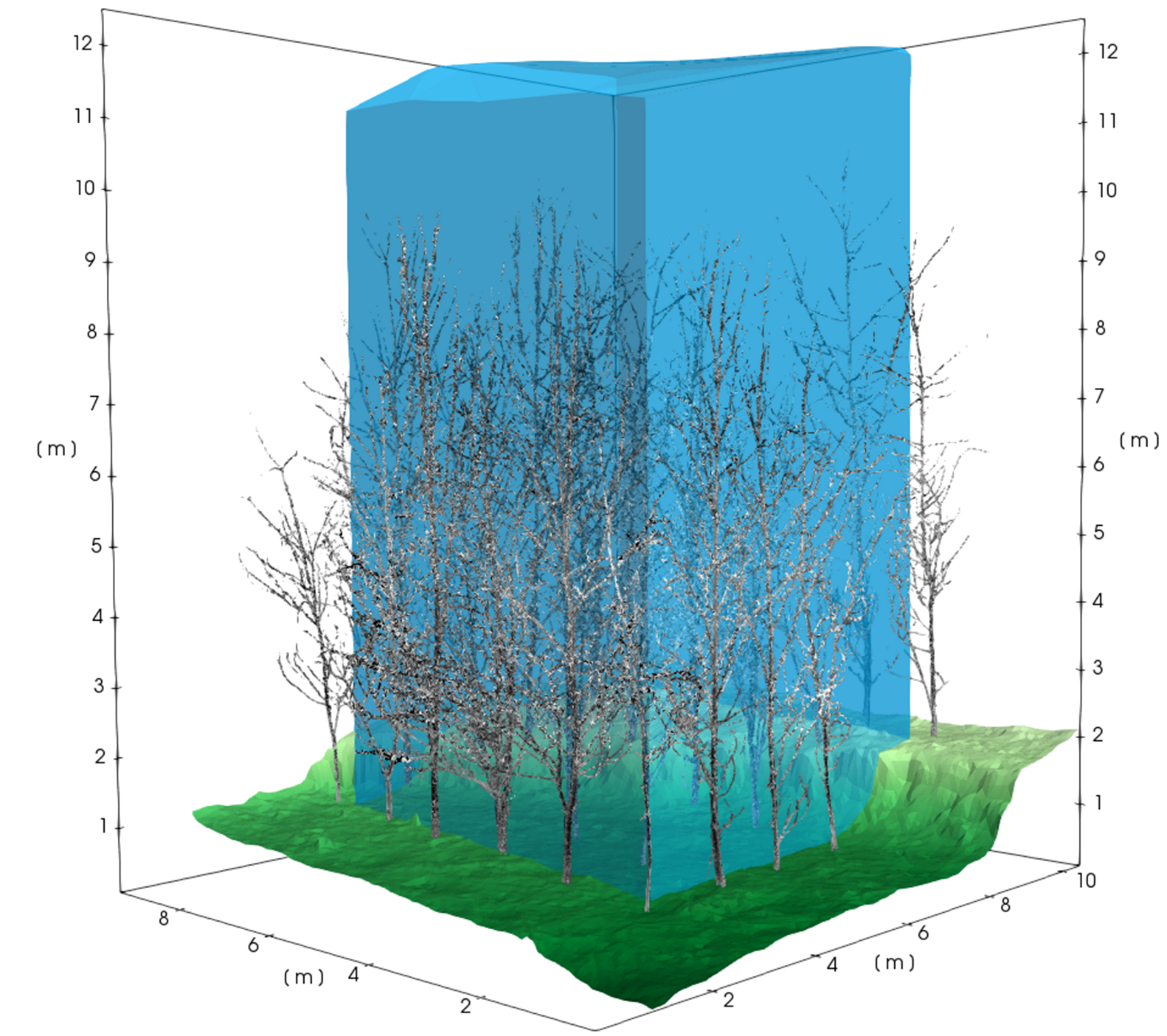


**Fig. S5**: Single tree point clouds and DTM of plot E34 scanned in 2015. The transparent blue polygon represents the investigation area, which is defined by a ground area of 6 m × 6 m and a height of 10.3 m above ground, resulting in a total space volume of 370.8 m^3^.


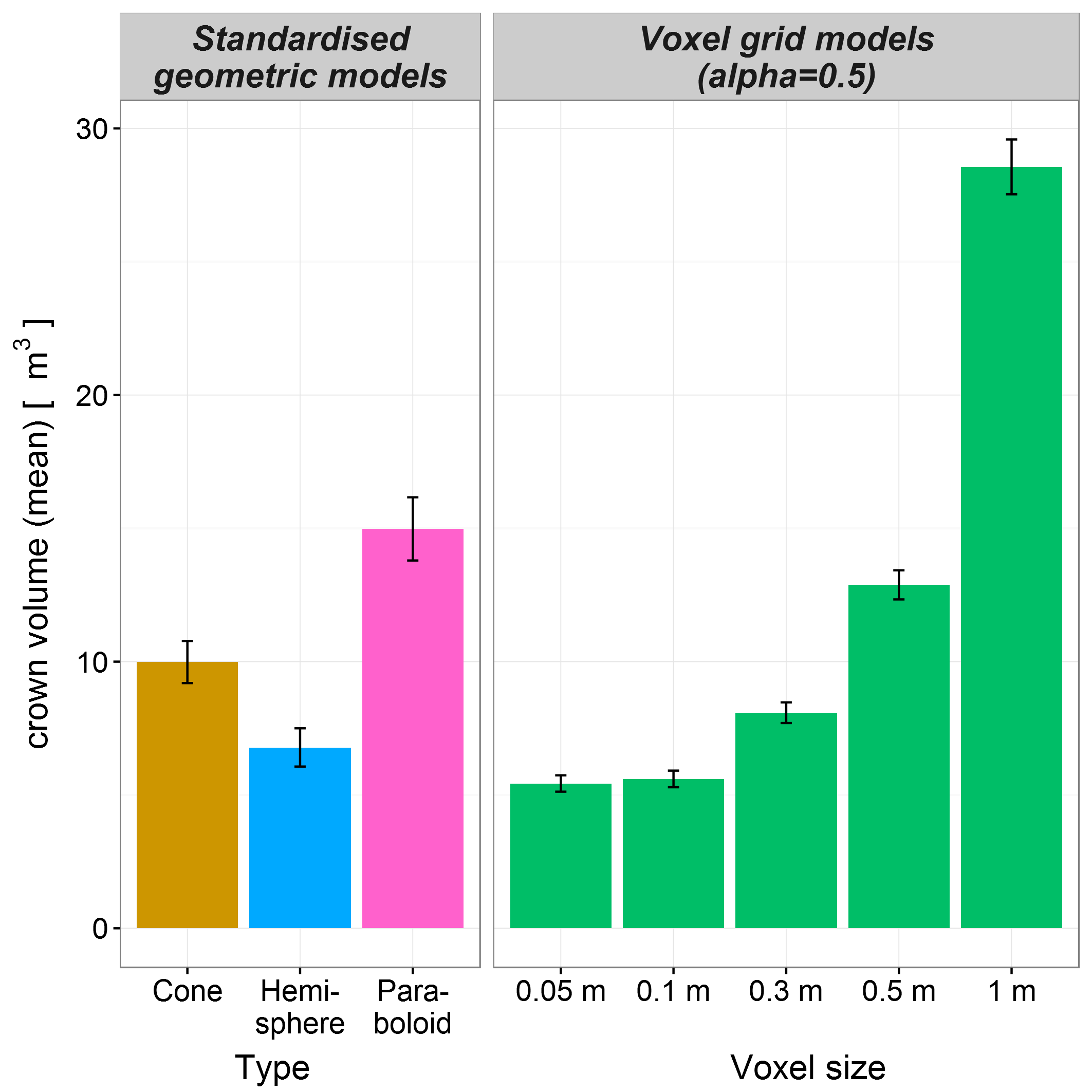


**Fig. S6**: Comparison of mean crown volumes (± SE) based on: standardised dendrometry crown models (Cone, Hemisphere, Parabolid) estimated from the inventory field measurement data (crown diameter, crown height) (left side); and TLS point cloud derived voxel grid crown models for five different voxel sizes (right side).
